# Transplantation outcomes in patients with primary hyperoxaluria: a systematic review

**DOI:** 10.1007/s00467-021-05043-6

**Published:** 2021-04-08

**Authors:** Elisabeth L. Metry, Liza M. M. van Dijk, Hessel Peters-Sengers, Michiel J.S. Oosterveld, Jaap W. Groothoff, Rutger J. Ploeg, Vianda S. Stel, Sander F. Garrelfs

**Affiliations:** 1 Department of Pediatric Nephrology, Emma Children’s Hospital, Amsterdam UMC, University of Amsterdam, Amsterdam, The Netherlands; 2 Center for Experimental and Molecular Medicine, Amsterdam UMC, University of Amsterdam, Amsterdam, The Netherlands; 3 Nuffield Department of Surgical Sciences, University of Oxford, Oxford, UK; 4 ERA-EDTA Registry, Department of Medical Informatics, Amsterdam Public Health Research Institute, Amsterdam UMC, University of Amsterdam, Amsterdam, The Netherlands

**Keywords:** Primary hyperoxaluria, liver transplantation, kidney transplantation, CKLT, SKLT, graft survival

## Abstract

**Background:**

Primary hyperoxaluria type 1 (PH1) is characterized by hepatic overproduction of oxalate and often results in kidney failure. Liver-kidney transplantation is recommended, either combined (CLKT) or sequentially performed (SLKT). The merits of SLKT and the place of an isolated kidney transplant (KT) in selected patients are unsettled. We systematically reviewed the literature focusing on patient and graft survival rates in relation to the chosen transplant strategy.

**Methods:**

We searched MEDLINE and Embase using a broad search string, consisting of the terms ‘transplantation’ and ‘hyperoxaluria’. Studies reporting on at least four transplanted patients were selected for quality assessment and data extraction.

**Results:**

We found 51 observational studies from 1975 to 2020, covering 756 CLKT, 405 KT and 89 SLKT, and 51 pre-emptive liver transplantations (PLT). Meta-analysis was impossible due to reported survival probabilities with varying follow-up. Two individual high-quality studies showed an evident kidney graft survival advantage for CLKT versus KT (87% vs. 14% at 15 years, *p*<0.05) with adjusted HR for graft failure of 0.14 (95% confidence interval: 0.05–0.41), while patient survival was similar. Three other high-quality studies reported 5-year kidney graft survival rates of 48–89% for CLKT and 14–45% for KT. PLT and SLKT yielded 1-year patient and graft survival rates up to 100% in small cohorts.

**Conclusions:**

Our study suggests that CLKT leads to superior kidney graft survival compared to KT. However, evidence for merits of SLKT or for KT in pyridoxine-responsive patients was scarce, which warrants further studies, ideally using data from a large international registry.

**Supplementary Information:**

The online version contains supplementary material available at 10.1007/s00467-021-05043-6.

## Introduction

The primary hyperoxalurias (PHs) are a group of rare inherited metabolic disorders leading to endogenous overproduction of oxalate. Three subtypes have been identified based on the underlying enzyme deficiency. PH type 1 (PH1) accounts for over 80% of all patients [1]. These patients present with kidney stones, nephrocalcinosis, or kidney failure in almost 40% of cases [2]. Eventually, over 70% will develop kidney failure [3]. In patients with advanced chronic kidney disease (CKD) or kidney failure, systemic oxalate storage occurs and causes multi-organ failure. Conservative therapy (e.g. hyperhydration and citrate supplementation to prevent stone formation) is not sufficient in those cases. So far, only liver transplantation can ‘cure’ the metabolic disorder and is therefore generally recommended in PH1 patients with kidney failure. A kidney transplantation is required since oxalate clearance by conventional dialysis cannot match endogenous oxalate production rate and thus will not prevent disease progression [4, 5].

Choosing the right transplantation strategy for each specific patient case remains challenging [4]. The most recent European guidelines recommend either combined liver-kidney transplantation (CLKT) or sequential liver-kidney transplantation (SLKT) in all PH1 patients with CKD stages 4 and 5 [6]. The guideline is reluctant to recommend pre-emptive liver transplantation (PLT), performed prior to the development of kidney failure and meant to prevent the need for dialysis and/or kidney transplantation. The procedure carries a significant mortality rate of 10% [7–9]. The guidelines further advise against isolated kidney transplantation (KT); it should be considered only for ‘selected adult patients with confirmed evidence of B6 responsiveness’ [6]. In approximately 30% of Western PH1 patients, vitamin B6 effectively lowers hepatic oxalate production. A small subset of patients shows a complete response defined as a normalization of oxalate excretion rate [3]. However, the paucity of data on performing an isolated KT in these patients prevents the guidelines from supporting such a deviation from the general recommendation in PH1 patients. As stated in the guideline, suggestions are based on ungraded statements because of the lack of randomized clinical trials and the rarity of PH1 [6]. It is of great clinical importance to identify the best transplantation strategy, as the entire transplantation procedure is costly and carries significant risks, including potentially fatal postoperative complications and the risk of tissue oxalate mobilization causing recurrent oxalate nephropathy in the kidney graft [6].

To our knowledge, this is the first systematic review of transplantation outcomes in PH. The aim of this systematic review was to compare patient and graft survival rates for different transplantation strategies in order to identify the optimal approach for PH patients. We feel that this will remain a relevant discussion, especially with new emerging therapies that appear to be effective but also may become very costly [10, 11].

## Methods

### Search strategy and study selection

This systematic review was conducted following the Preferred Reporting Items for Systematic reviews and Meta-analyses (PRISMA) guidelines and PROSPERO statement [12]. The electronic databases MEDLINE (PubMed) and Embase (Ovid) were searched for relevant literature to identify studies examining transplantation outcomes in PH patients who underwent transplantation. The search was run on November 19, 2019, and repeated on November 11, 2020. We used a broad search string, consisting of the terms ‘hyperoxaluria and transplantation’. The full search strategy is provided in Supplementary Table 1. No restrictions on article type, publication status, publication year and language were set. Screening of title and abstract was performed independently by two reviewers (LMD and SFG). Studies were eligible if the authors described PH patients who received any organ transplant and reported on patient and/or graft survival following CLKT, SLKT, KT or LT in adult or paediatric subjects. In case title and abstract did not provide sufficient information on these keywords, articles were included for full-text screening as well. If various publications of the same data were available, only the most recent study was assessed for eligibility. This only concerned studies that described the exact same patient cohort over time. Two reviewers (LMD and ELM) individually screened the full text of possible relevant papers. Any disagreement was resolved in discussion with the third reviewer (SFG). The diagnosis of PH1 needed to be based on either mutation analysis, liver biopsy, clinical phenotype with secondary causes of hyperoxaluria excluded or disease code in a registry. We excluded patients who were only diagnosed after transplantation since the diagnosis of PH had not been taken into account in deciding transplantation strategy nor in the pre-transplantation work-up and treatment. Case reports, case series that included less than four patients, conference abstracts and articles without original data were excluded as well. If the patient cohort consisted of both PH patients and other transplant recipients, outcomes had to be described separately for the PH patients.

### Quality assessment and data extraction

Methodological quality was determined using a modified version of the Downs and Black Checklist (see Supplementary Table 2). This checklist was considered the best instrument for systematic reviews that included different study designs in terms of reliability and validity and frequency of use in the literature [13]. The checklist assesses the risk of bias in five categories: reporting, external validity, internal validity—bias, internal validity—confounding and power. Concerning the confounding category, items 21 and 22, we assigned a point to studies that compared two different interventions, performed in patients from the same population and over the same period of time. We did not assign a point to studies comparing outcomes in PH patients with outcomes in patients with other diagnoses. Concerning the power category, we modified item 27 (one point for carrying out a power calculation), as proposed by Kennelly et al. [14]. Therefore, instead of the original 32, the highest possible total score was 28. Also according to Kennelly, we considered the quality of the study to be good if the study scored at least 20 points, fair with a score of 15–19 points and poor with a score of 14 points or lower.

Two authors (LMD and ELM) extracted the following data: characteristics of included studies (first author, year of publication, inclusion period of the study, number of transplanted patients), characteristics of eligible patients (sex and age at transplantation, type and duration of pre-transplant dialysis and transplantation type) and transplantation outcomes (follow-up duration, patient survival, graft survival, graft loss, cause of graft loss, estimated glomerular filtration rate (eGFR)).

## Results

### Study characteristics

**Fig. 1:**
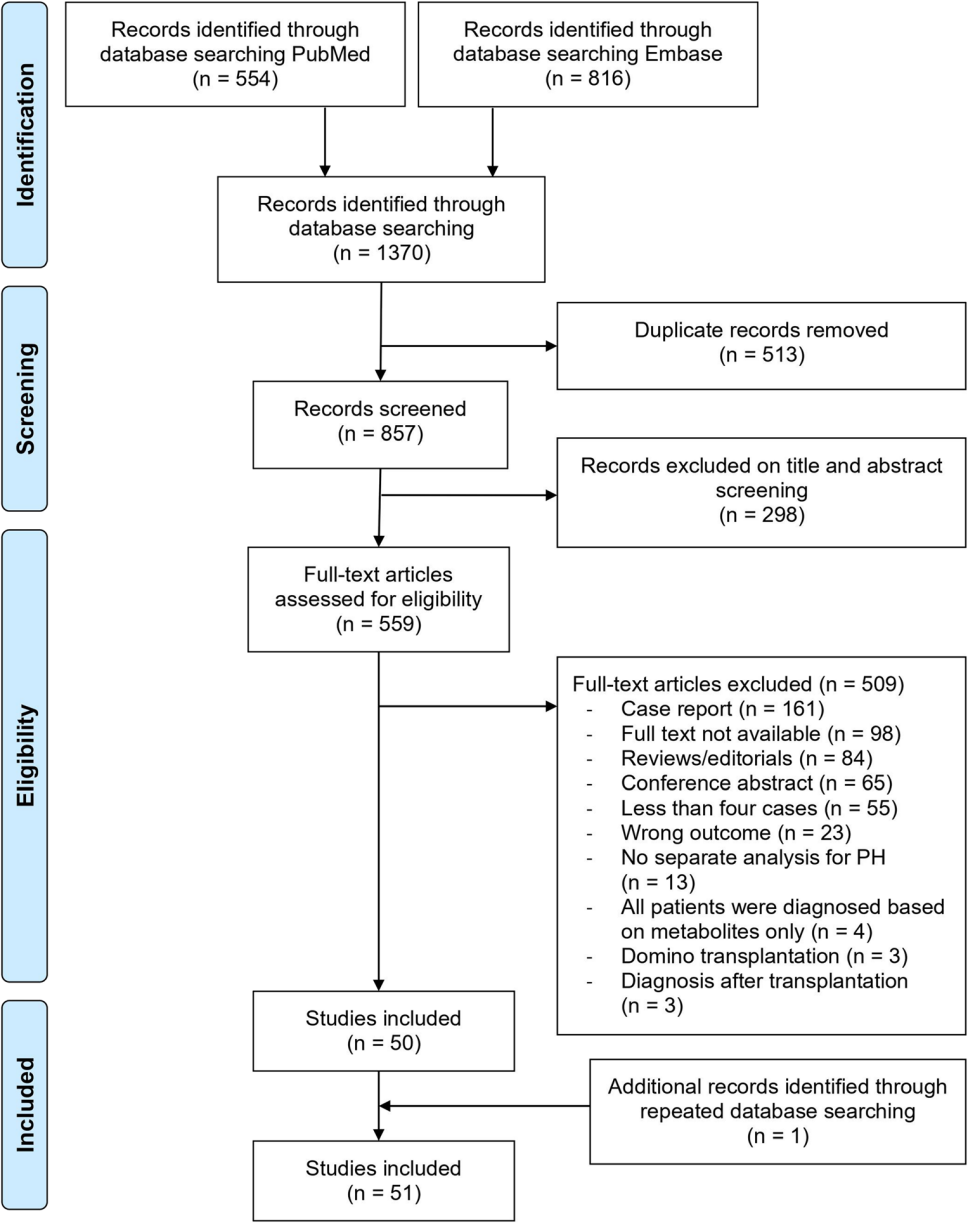
The study selection procedure

We included 51 observational studies (see Fig. 1 for the study selection procedure). We found nine registry studies [4, 15–22], 35 single-centre studies [8, 23–56], two multicentre studies [57, 58] and five studies using questionnaires [9, 59–62]. The studies were published between 1975 and 2020, and the number of included PH1 patients ranged from four to 201 (median 8, IQR 5–24). Only 12 PH2 patients received a transplantation [17, 43]; therefore, they were not included in any further analysis. There were no reports on transplantations in PH3 patients. In 1201 PH1 patients, 756 CLKTs, 405 KTs, 89 SLKTs and 51 PLTs were performed. In 37 studies, the diagnosis of PH1 was established by mutation analysis or liver biopsy. In the other studies, the diagnosis was based on clinical phenotype (e.g. hyperoxaluria with secondary causes excluded) or disease code in registries.

**Fig. 2:**
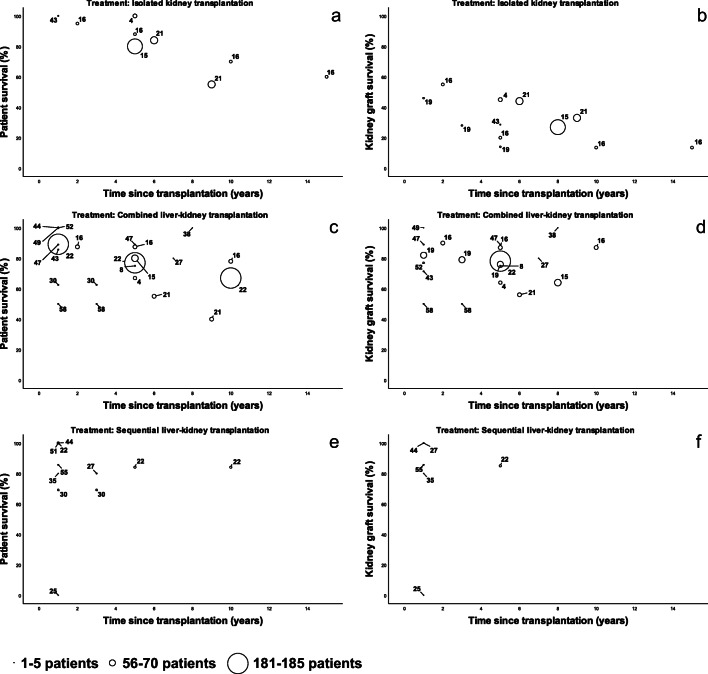
Reported patient (**a**, **c**, **e**) and kidney graft survival (**b**, **d**, **f**) in the included studies according to transplantation strategy. Circle size represents the size of the described cohorts; numbers refer to the references, not sample size

According to this modified Downs and Black Checklist, nine studies were of strong quality. Twenty-two studies were assessed as being of moderate quality, and twenty studies were of low quality (Supplementary Table 3). Meta-analyses were considered inappropriate because included studies reported survival probabilities with varying follow-up durations instead of relative risks. Therefore, studies of strong quality that compared different transplantation techniques in PH1 patients will be discussed in detail; characteristics and outcomes of all studies are provided in Supplementary Tables 4, 5 and 6 and Fig. 2.

### Combined liver-kidney transplant versus isolated kidney transplant

Table 1 summarizes the results of five studies of strong quality that made an attempt to compare the outcomes of CLKT to KT in PH patients [4, 15, 16, 19, 43]. Only two studies reported hazard ratios [16, 19]. A registry study by Bergstralh et al. was the only study to report a difference in patient survival in favour of KT, since three patients died following CLKT [4]. Patients died with a functioning graft which explains the difference between 5-year kidney graft survival (48% versus 45% for CLKT as compared to KT) and 5-year death-censored kidney graft survival, which was in favour of CLKT (71% versus 45%). Three other multicentre or registry studies found a significantly better (death-censored) kidney graft survival for CLKT [15, 16, 19]. This remained true, analysing outcomes separately for recipient age (younger or older than 15 years) and year of transplantation (prior to or after 1995) [14]. Finally, the occurrence of postoperative severe complications (grades III and IV according to the classification of Dindo et al. [63]) was similar for CLKT (48.0%) and KT (51.0%) [16]. None of the above studies reported if patients were responsive to vitamin B6.

**Table 1:** Strong quality studies comparing combined liver–kidney transplantation with (1) isolated kidney transplantation and (2) sequential liver–kidney transplantation

Reference (country)	Journal	Year of publication	Inclusion period	Adults/ children	Number of patients	Patient survival	Kidney graft survival	Conclusion paper
**CLKT/KT**								
Monico [43] (U.S.A.)	Liver Transpl	2001	1968–2000	Adults	7/8	2.1 y 71%/ 1.8 y 88%	1 y 71%/ 5 y 29%	No conclusions made
Compagnon [16] (France)	Liver Transpl	2014	1979–2010	Both	33/21	15 y 78%/ 15 y 60% (*p* = 0.49, HR 1.45, 95% CI 0.5–4.1)	DC-KGS 10 y 87%/ 10 y 13% (*p* < 0.001, HR 8.6, 95% CI 53.3–22.2)	Better DC-KGS for CLKT
Harambat [19] (France)	Clin J Am Soc Nephrol	2012	1979–2009	Children	55/13^c^	No data^c^	5 y 76%/ 5 y 14%^d^	Better kidney graft survival for CLKT
Bergstralh [4] (U.S.A.)	Am J Transplant	2010	1976–2009	Adults	26/32	5 y 67%/ 5 y 100% (p = 0.035)	5 y 48%/ 5 y 45% (p = 0.137, 5 y DC-KGS 71% / 45%^a^, p = 0.011)	Better DC-KGS for CLKT
Cibrik [15] (U.S.A.)	Transplantation	2002	1988–1998	Adults	56/134	8 y 66%/ 8 y 67%	DC-KGS^b^ 8 y 76%/ 8 y 47.9% (*p* < 0.001)	Better DC-KGS for CLKT
**CLKT/SLKT**								
Xiang [22] (China; data from U.S.A.)	BMC Gastroenterol	2020	1987–2018	Both	181/20	10 y 67%/ 10 y 84% (*p* = 0.717)	5 y 78%/ 5 y 85% (*p* = 0.464)	SLKT is a viable alternative treatment to CLKT
Horoub [30] (Iran)	Exp Clin Transplant Assoc	2019	2011–2018	Both	8/13	3 y 62%/ 3 y 69% (*p* > 0.05)	3 y 62%/ 3 y 69% (*p* > 0.05)	No significant differences
Büscher [27] (Germany)	Pediatr Transplant	2015	1998–2013	Children	5/6	7 y 80%/ 3 y 80%, 10 y 76%	7 y 80% 1 y 100%	Good outcomes for both CLKT and SLKT in children

DC-KGS = death-censored kidney graft survival; HR = hazard ratio

^a^84 transplantations in 58 patients of which 32/26 first CLKT/KT

^b^adjusted for multiple covariates: recipient age, race, and gender; repeat transplants; immunosuppression; cytomegalovirus; donor source (cadaveric vs. living), race and age; KT and LKT; cold ischemic time; panel reactive antibody; HLA mismatch; time on dialysis; and year of transplantation

^c^53 combined, 2 sequential. Patient survival after commencing kidney replacement therapy: 5y 83% (2000-2009) and 71% (before 2000), no data on difference CLKT/KT

^d^adjusted for age, sex and decade of start of KRT

### Combined liver-kidney transplant versus sequential liver-kidney transplant

Three studies of strong quality compared patient and kidney graft survival rates between CLKT and SLKT recipients [22, 27, 30]. None of the studies found a significant difference in patient or kidney graft survival when comparing CLKT to SLKT. Recently, Xiang et al. reported on the highest number of patients (*n* = 20) undergoing SLKT [22]. Of note, an unknown number of patients who were scheduled for SLKT but died after liver transplantation were not included in this registry study. Horoub et al. studied a cohort with high mortality rates in both groups [30]. All patients received a liver and kidney transplant. Causes of death were primary graft nonfunction, massive gastrointestinal bleeding, multi-organ failure, sepsis and cerebrovascular accident. All surviving patients had a functioning graft at last follow-up with similar mean GFR (CKD stage 2 for both groups, *p* = 0.201). More favourable results were reported in a German study with paediatric patients [27]. Ten-year patient survival was 75.8% without any significant differences between the transplantation strategies. All surviving CLKT patients had a functioning kidney graft after a median follow-up of 11.8 years (range 7.0–16.3). One patient died due to fibrosis of portal vein thrombosis after receiving both a liver and kidney transplant. Two SLKT patients had not received a kidney transplant yet; the three remaining surviving patients had good functioning kidney grafts at a median follow-up of 3.2 years (1.5–12.7). In nineteen moderate and low-quality studies, a total of fifty patients underwent sequential liver transplantation, of whom eight patients died prior to kidney transplantation.

### Isolated kidney transplant in pyridoxine-responsive patients

Only one study provided information on isolated kidney transplantations in pyridoxine-responsive PH1 patients. In a small case series, Lorenz et al. investigated kidney transplant outcomes of four adults with PH1, who were homozygous for G170R mutation [37]. Age at symptoms ranged from 6–37 years. They developed stage 5 CKD at the age of 33–67 and underwent transplantation in the same year. The patients had been treated with pyridoxine (5–8 mg/kg/day) prior to transplantation, effectively lowering urinary oxalate values. Urine oxalate excretion remained normal or near normal (< 0.5 mmol/24h) on 33/50 follow-up visits. At a median follow-up of 5.2 years (range 0.2–13.9), all four kidney grafts were functioning with eGFR 34–57 mL/min/1.73 m^2^.

### Pre-emptive liver transplantation

No studies of strong quality compared outcomes of PLT to another transplantation strategy. Brinkert et al. included the highest number of four patients and reported on a 100% patient and graft survival after 10 years [25]. In total, 51 patients received a pre-emptive liver transplant of whom outcomes were described in only 34 cases. Median age at transplantation was 5 years (range 10 months to 23 years). Out of 34 liver transplantations, 30 were functioning at time of last follow-up (median 4 years, range 1–16 years). Liver graft failure was reported in two cases [30, 48] and three patients died [30, 60, 61]. Kidney function stabilized in 25 cases following transplantation, and at least ten of them showed improved kidney function (range 20.0–43.8%, Table 2). Four patients progressed to stage 5 CKD and underwent KT during follow-up; three of them after more than 5 years post-PLT.

**Table 2:** Mean kidney function following pre-emptive liver transplantation

Reference	Patients (*n*)	Mean GFR pre-transplantation [ml/min/1.73 m^2^]	Mean GFR post-transplantation [ml/min/1.73 m^2^]	Improvement [%]
Horoub [30]	3	58.4^a^	84.0^a^	43.8
Shapiro [53]	1	-	-	20
Brinkert [25]	3	78	104.7	34.2
Khorsandi [35]	3	38.7	55	42.1

^a^ml/min

## Discussion

We systematically reviewed outcomes of different transplant modalities used in PH1. In total, we identified 51 observational studies on transplantation outcomes in 1201 PH1 patients. Outcomes were mainly reported as survival probabilities; only two studies reported hazard ratios [16, 19]. Out of five high-quality studies, only one study found a statistically significant difference in patient survival, in favour of KT [4]. In this study however, outcomes were not adjusted for year of transplantation or any other factors. In previous decades, CLKT was a ‘hazardous venture’ [21]. There were no significant differences in patient survival at 15 years post-transplantation (78% for CLKT and 60% for KT) according to a multicentre study by Compagnon et al. [16]. The same was observed in a large registry study by Cibrik et al. [15]. Both studies adjusted for several factors including year of transplantation. The risk of death due to complications of the procedure for CLKT seems to have outbalanced the risk of death due to severe oxalosis in KT recipients in the long term.

The substantially higher kidney graft survival for CLKT recipients (87% at 10 years [16]) is expected to be due to the pathophysiology of PH; the devastating kidney graft survival rates for KT (14% at 10 years [16]) can be ascribed to the unabated hepatic oxalate production and release of stored oxalate, resulting in damage to the kidney transplant soon after the procedure. However, genotype and clinical pyridoxine responsiveness are of major importance with regard to the risk of graft failure in these patients but were not reported in all except one study.

In the case series by Lorenz et al., four pyridoxine-responsive patients successfully received a KT in combination with conservative therapy [37]. eGFR was moderately reduced (CKD stage 3) at a median follow-up of 5.2 years (range 0.2–13.9). In a large cohort of non-PH kidney transplant recipients, the distribution of CKD stages 1–5 at 12 months was 2.7, 27.1, 59.4, 10.3 and 0.5%. This was very similar at 5 years and 10 years of follow-up [64]. The idea that KT may be a viable option in this subgroup of (adult) patients who are deemed to be completely responsive to pyridoxine and are expected to have better outcomes [65] has been suggested previously [6, 66]. Despite the good clinical reasoning behind performing a KT in patients who clinically respond to pyridoxine therapy, there is a lack of evidence to support this approach and consequently clinicians opt for a liver-kidney transplant.

The merits of SLKT as compared to CLKT are not evident, mainly due to the small number of studies comparing both strategies (maximum 20 SLKT procedures [20]). Sequential procedures were performed in patients with severe systemic oxalosis [46] and small infants [27]. Even at the age of 4 months, an infant successfully underwent liver transplantation and is now awaiting a kidney transplant [27]. Also, SLKT has been performed safely with organs retrieved from a single living donor [46, 55]. In that case, this strategy attains the immunological advantage of a CLKT. However, in most cases, two donors are needed for a SLKT procedure [67]. Very few cases of SLKT have been reported and even fewer reports of donor outcomes exist. Therefore, the guidelines do not favour either CLKT or SLKT and advise on a simultaneous or sequential procedure according to the patient’s condition, local facilities and preferences [6].

Pre-emptive liver transplantations were not widely performed, but case series reported very high patient survival rates, up to 100% after 10 years of follow-up [25]. However, the European Liver Transplant Registry (ELTR) reported a 1-y mortality rate of 16% for 258 PH patients who underwent liver transplantation between 2001 and 2016 (presumably combined with a kidney transplant in most cases) [68]. Even while children affected with metabolic disorders are known to achieve the best outcomes [68], their mortality rates remain considerably high, and this holds true for transplantations performed in the past two decades. Death due to long-term complications of chronic usage of immunosuppressive medication should be added onto that. A review by Kemper et al. included nine patients who received a pre-emptive liver transplant of whom four patients required either a second liver transplant or a kidney transplant during follow-up [69]. Yet, differences in follow-up duration hamper a valid comparison between studies. The current guidelines do therefore not recommend this approach considering the ethical dilemma of performing a risky procedure in a patient who could remain stable for many years with conservative treatment only [6].

The most important limitations of this systematic review are due to the observational nature of the included studies, in which confounding by indication plays a role. Even in the few high-quality studies that did correct for confounders, residual confounding cannot be excluded. A meta-analysis could not be performed since survival probabilities were reported with various follow-up durations. Recently, methods have been developed to reconstruct time-to-event data from published Kaplan–Meier curves [70]. However, the few high-quality studies differed in patient population in terms of period of time, country and pre-transplant care, to the extent that an attempt to pool these heterogeneous data was considered inappropriate. Additionally, a comparison between CLKT and KT in pyridoxine-responsive patients was not feasible due to the lack of reporting of genotypes. Determinants of graft failure or death are rarely studied in PH patients; only Cibrik et al. assessed the influence of covariates and found multiple transplants, recipient ethnicity, panel reactive antibody, cold ischemic time and donor age as significant risk factors for death-censored kidney graft survival [15]. Furthermore, some included studies based their diagnosis on clinical phenotype, not liver biopsy or mutation analyses. Even relatively recently performed high-quality studies included patients whose diagnoses were based on metabolites only [4, 16]. Also, publication bias is likely to play a role in our review. This is rather true because researchers tend to publish nice-ending small studies of their transplantations, especially considering pre-emptive liver transplantations, which could explain the 100% 1-y survival rate in studies solely describing outcomes of this type of transplant. As a final limitation, we cannot exclude that there was any overlap of included patients. We excluded studies that evidently described the same patient cohort in a previous period of time, but individual patients may have been registered in more than one registry and thus described in more than one study. It is unlikely that this would concern a substantial number of studies since most included studies were single-centre studies.

The findings of this systematic review suggest that a combined or sequential liver-kidney transplantation has to be recommended as the first choice for treatment of PH1. This conclusion is however based on a relatively small number of cohort studies and registry studies that were of relatively good quality, which do not capture important patient characteristics such as genotype. Due to the rareness of this disease and the impossibility of performing randomized controlled trials, a well-maintained international registry is crucial for comparing outcomes for different transplantation strategies. This is needed to demonstrate possible merits of SLKT. In particular, there is a great need for studies investigating the possibility of KT in pyridoxine-responsive patients. The spectrum of therapeutic options to treat PH is expected to be expanded in the near future: medications comprising small interference RNA are emerging. Indeed, the investigational product Oxlumo has recently been approved for all ages by both the EMA and FDA as the first pharmaceutical treatment for PH1 [71, 72]. Preliminary data by Alnylam pharmaceuticals show that urinary oxalate excretion is effectively lowered with 65% and 72% mean reduction relative to baseline, in adults and children, respectively. Promising results are presented by Dicerna pharmaceuticals as well; due to the different mechanisms of action, these clinical trials also include patients with primary hyperoxaluria type 2 and 3 (clinicaltrialsgov, NCT number 03847909). These new medications will likely obviate the need for a liver transplant, but at first this will not be available for everyone. In addition, a kidney transplant will remain inevitable for patients who have already proceeded to CKD stage 5 [73]. This systematic review provides an overview of transplantation approaches in order to contribute to evidence-based decision-making. Yet, it also identifies the knowledge gap concerning outcomes of kidney transplants in pyridoxine-responsive patients, who might benefit from an isolated kidney transplant even in this new era. The rarity of PH1 should encourage close cooperation between expert PH centres to fill that gap and identify the optimal transplantation strategy for individual patients that will further enhance survival and quality of life of PH1 patients.

## Supplementary Information

ESM 1 (DOCX 78 kb)

## Data Availability

All data generated during this study are included in this article and its supplementary files.
