# Supplementary material for: Transplantation outcomes in patients with primary hyperoxaluria: a systematic review
Source: Pediatr Nephrol. 2021 Apr 8;36(8):2217–26. doi: 10.1007/s00467-021-05043-6 (PMC8260423; doi:10.1007/s00467-021-05043-6)
Supplement: Supplementary file 1 — (DOCX 78 kb) [file 467_2021_5043_MOESM1_ESM.docx]

**Supplementary Table 1.**

**Search Strategy**

| Electronic database | Search string | Number of articles found on  Nov 19^th^ 2019 |
| --- | --- | --- |
| MEDLINE (PubMed) | ("Hyperoxaluria"[Mesh] OR Hyperoxaluria[Title/Abstract] OR Hyperoxaluria OR Oxaluria[Title/Abstract] OR Oxaluria)  AND  ("Transplantation"[Mesh] OR Transplant*[Title/Abstract] OR Graft*[Title/Abstract]) | 554 |
| Embase (OVID) | (Hyperoxaluria.ti,ab,kw. OR Hyperoxaluria.af. OR Hyperoxaluria/ OR Oxaluria.ti,ab,kw. OR Oxaluria.af.)  AND  (Transplant*.ti,ab,kw. OR Transplantation/ OR Graft*.ti,ab,kw.) | 816 |

**Supplementary Table 2.**

**Modified Downs and Black checklist for the assessment of the methodological quality of both randomized and non-randomized studies^1^**

| Item | Criteria | Possible answers |
| --- | --- | --- |
| Reporting | | |
| 1 | *Is the hypothesis/aim/objective of the study clearly described?* | Yes = 1  No = 0 |
| 2 | *Are the main outcomes to be measured clearly described in the Introduction or Methods section?*  If the main outcomes are first mentioned in the Results section, the question should be answered no. | Yes = 1  No = 0 |
| 3 | *Are the characteristics of the patients included in the study clearly described?*  In cohort studies and trials, inclusion and/or exclusion criteria should be given. In case-control studies, a case-definition and the source for controls should be given. | Yes = 1  No = 0 |
| 4 | *Are the interventions of interest clearly described?*  Treatments and placebo (where relevant) that are to be compared should be clearly described. | Yes = 1  No =0 |
| 5 | *Are the distributions of principal confounders in each group of subjects to be compared clearly described?*  A list of principal confounders is provided. | Yes = 2  Partially = 1  No = 0 |
| 6 | *Are the main findings of the study clearly described?*  Simple outcome data (including denominators and numerators) should be reported for all major findings so that the reader can check the major analyses and conclusions. (This question does not cover statistical tests which are  considered below). | Yes = 1  No = 0 |
| 7 | *Does the study provide estimates of the random variability in the data for the main outcomes?*  In non-normally distributed data the interquartile range of results should be reported. In normally distributed data the standard error, standard deviation or confidence intervals should be reported. If the distribution of the data is not described, it must be assumed that the estimates used were appropriate and the question should be answered yes. | Yes = 1  No = 0 |
| 8 | *Have all important adverse events that may be a consequence of the intervention been reported?*  This should be answered yes if the study demonstrates that  there was a comprehensive attempt to measure adverse  events. (A list of possible adverse events is provided). | Yes = 1  No = 0 |
| 9 | *Have the characteristics of patients lost to follow-up been*  *described?*  This should be answered yes where there were no losses to  follow-up or where losses to follow-up were so small that  findings would be unaffected by their inclusion. This should be answered no where a study does not report the number of patients lost to follow-up. | Yes = 1  No = 0 |
| 10 | *Have actual probability values been reported (e.g. 0.035*  *rather than <0.05) for the main outcomes except where the*  *probability value is less than 0.001?* | Yes = 1  No = 0 |
| External validity | | |
| 11 | *Were the subjects asked to participate in the study*  *representative of the entire population from which they were*  *recruited?*  The study must identify the source population for patients  and describe how the patients were selected. Patients would  be representative if they comprised the entire source  population, an unselected sample of consecutive patients, or  a random sample. Random sampling is only feasible where  a list of all members of the relevant population exists. Where  a study does not report the proportion of the source  population from which the patients are derived, the question  should be answered as unable to determine. | Yes = 1  No = 0  Unable to  determine = 0 |
| 12 | *Were those subjects who were prepared to participate*  *representative of the entire population from which they were*  *recruited?*  The proportion of those asked who agreed should be stated. Validation that the sample was representative would include  demonstrating that the distribution of the main confounding  factors was the same in the study sample and the source  population. | Yes = 1  No = 0  Unable to  determine = 0 |
| 13 | *Were the staff, places, and facilities where the patients were*  *treated, representative of the treatment the majority of*  *patients receive?*  For the question to be answered yes the study should  demonstrate that the intervention was representative of that  in use in the source population. The question should be  answered no if, for example, the intervention was  undertaken in a specialist centre unrepresentative of the  hospitals most of the source population would attend. | Yes = 1  No = 0  Unable to  determine = 0 |
| Internal validity - bias | | |
| 14 | *Was an attempt made to blind study subjects to the intervention they have received?*  For studies where the patients would have no way of knowing which intervention they received, this should be answered yes. | Yes = 1  No = 0  Unable to determine = 0 |
| 15 | *Was an attempt made to blind those measuring the main outcomes of the intervention?* | Yes = 1  No = 0  Unable to determine = 0 |
| 16 | *If any of the results of the study were based on “data*  *dredging”, was this made clear?*  Any analyses that had not been planned at the outset of the  study should be clearly indicated. If no retrospective  unplanned subgroup analyses were reported, then answer  yes. | Yes = 1  No = 0  Unable to  determine = 0 |
| 17 | *In trials and cohort studies, do the analyses adjust for*  *different lengths of follow-up of patients, or in case-control*  *studies, is the time period between the intervention and*  *outcome the same for cases and controls?*  Where follow-up was the same for all study patients the  answer should be yes. If different lengths of follow-up were adjusted for by, for example, survival analysis the answer should be yes. Studies where differences in follow-up are ignored should be answered no. | Yes = 1  No = 0  Unable to  determine = 0 |
| 18 | *Were the statistical tests used to assess the main outcomes*  *appropriate?*  The statistical techniques used must be appropriate to the  data. For example nonparametric methods should be used  for small sample sizes. Where little statistical analysis has  been undertaken but where there is no evidence of bias, the  question should be answered yes. If the distribution of the  data (normal or not) is not described it must be assumed  that the estimates used were appropriate and the question  should be answered yes. | Yes = 1  No = 0  Unable to  determine = 0 |
| 19 | *Was compliance with the intervention/s reliable?*  Where there was noncompliance with the allocated  treatment or where there was contamination of one group,  the question should be answered no. For studies where the  effect of any misclassification was likely to bias any  association to the null, the question should be answered  yes. | Yes = 1  No = 0  Unable to  determine = 0 |
| 20 | *Were the main outcome measures used accurate (valid and*  *reliable)?*  For studies where the outcome measures are clearly  described, the question should be answered yes. For  studies which refer to other work or that demonstrates the  outcome measures are accurate, the question should be  answered as yes. | Yes = 1  No = 0  Unable to  determine = 0 |
| Internal validity – confounding (selection bias) | | |
| 21 | *Were the patients in different intervention groups (trials and*  *cohort studies) or were the cases and controls (case-control*  *studies) recruited from the same population?*  For example, patients for all comparison groups should be  selected from the same hospital. The question should be  answered unable to determine for cohort and case-control  studies where there is no information concerning the source  of patients included in the study. | Yes = 1  No = 0  Unable to  determine = 0 |
| 22 | *Were study subjects in different intervention groups (trials*  *and cohort studies) or were the cases and controls (case-*  *control studies) recruited over the same period of time?*  For a study which does not specify the time period over  which patients were recruited, the question should be  answered as unable to determine. | Yes = 1  No = 0  Unable to  determine = 0 |
| 23 | *Were study subjects randomized to intervention groups?*  Studies which state that subject were randomized should be answered yes except where method of randomization would not ensure random allocation. For example alternate allocation would score no because it is predictable. | Yes = 1  No = 0  Unable to determine = 0 |
| 24 | *Was the randomization intervention assignment concealed from both patients and health care staff until recruitment was complete and irrevocable?* | Yes = 1  No = 0  Unable to determine = 0 |
| 25 | *Was there adequate adjustment for confounding in the*  *analyses from which the main findings were drawn?*  This question should be answered no for trials if: the main  conclusions of the study were based on analyses of  treatment rather than intention to treat; the distribution of  known confounders in the different treatment groups was not  described; or the distribution of known confounders differed between the treatment groups but was not taken into account in the analyses. In non-randomized studies if the effect of the main confounders was not investigated or confounding was demonstrated but no adjustment was made in the final analyses the question should be answered as no. | Yes = 1  No = 0  Unable to  determine = 0 |
| 26 | *Were losses of patients to follow-up taken into account?*  If the numbers of patients lost to follow-up are not reported,  the question should be answered as unable to determine. If  the proportion lost to follow-up was too small to affect the  main findings, the question should be answered yes. | Yes = 1  No = 0  Unable to  determine = 0 |
| Power | | |
| 27^a^ | *Did the study have sufficient power to detect a clinically*  *important effect where the probability value for a difference*  *being due to chance is less than 5%?*  Sample sizes have been calculated to detect a difference of  x% and y%. | Yes = 1  No = 0  Unable to  determine = 0 |

^a^ Item has been modified.

**Reference**

1. Downs SH, Black N. The feasibility of creating a checklist for the assessment of the methodological quality both of randomised and non-randomised studies of health care interventions. J Epidemiol Community Health. Jun 1998;52(6):377-84. doi:10.1136/jech.52.6.377

**Supplementary Table 3.**

**Critical Appraisal Score According to the Downs and Black Checklist**

| Reference | Reporting (maximum score = 11) | External validity (maximum  score = 3) | Internal validity – bias (maximum score = 7) | Internal validity – confounding (maximum score = 6) | Power (maximum score = 1) | Total score (maximum score = 28) |
| --- | --- | --- | --- | --- | --- | --- |
| Strong (20-28 points) | | | | | | |
| Monico [43] | 11 | 3 | 5 | 4 | 0 | **23** |
| Compagnon [16] | 10 | 3 | 5 | 4 | 0 | **22** |
| Harambat [19] | 10 | 3 | 5 | 4 | 0 | **22** |
| Bergstralh [4] | 11 | 3 | 5 | 3 | 0 | **22** |
| Xiang [22] | 11 | 3 | 4 | 3 | 0 | **21** |
| Cibrik [15] | 9 | 3 | 5 | 4 | 0 | **21** |
| Horoub [30] | 9 | 3 | 5 | 3 | 0 | **20** |
| Büscher [27] | 10 | 3 | 4 | 3 | 0 | **20** |
| Perera [47] | 11 | 3 | 5 | 1 | 0 | **20** |
| Moderate (15-19 points) | | | | | |  |
| Schmaeschke [52] | 10 | 3 | 4 | 2 | 0 | **19** |
| Nissel [57] | 10 | 3 | 4 | 2 | 0 | **19** |
| Shapiro [53] | 10 | 3 | 5 | 1 | 0 | **19** |
| Quintero Bernabeu [50] | 9 | 3 | 5 | 1 | 0 | **18** |
| Perera [48] | 10 | 3 | 4 | 1 | 0 | **18** |
| Millan [41] | 9 | 3 | 5 | 1 | 0 | **18** |
| Garrelfs [17] | 8 | 3 | 5 | 1 | 0 | **17** |
| Mantel [40] | 8 | 3 | 5 | 1 | 0 | **17** |
| Brinkert [25] | 9 | 3 | 3 | 1 | 0 | **16** |
| Ozer [46] | 9 | 3 | 3 | 1 | 0 | **16** |
| Duclaux-Loras [28] | 9 | 3 | 3 | 1 | 0 | **16** |
| Khorsandi [35] | 9 | 3 | 3 | 1 | 0 | **16** |
| Gagnadoux [8] | 7 | 3 | 5 | 1 | 0 | **16** |
| Pratschke [49] | 7 | 3 | 5 | 1 | 0 | **16** |
| Saborio [21] | 7 | 3 | 5 | 1 | 0 | **16** |
| Watts [62] | 8 | 3 | 4 | 1 | 0 | **16** |
| Kotb [36] | 8 | 2 | 4 | 1 | 0 | **15** |
| Sasaki [51] | 8 | 3 | 3 | 1 | 0 | **15** |
| Lorenzo [38] | 8 | 3 | 3 | 1 | 0 | **15** |
| Jamieson [20] | 6 | 3 | 5 | 1 | 0 | **15** |
| Creput [56] | 8 | 3 | 4 | 0 | 0 | **15** |
| Milliner [42] | 8 | 3 | 3 | 1 | 0 | **15** |
| Low (0-14 points) | | | | | | |
| Kitajima [55] | 6 | 3 | 4 | 1 | 0 | **14** |
| Al Riyami [23] | 6 | 3 | 3 | 2 | 0 | **14** |
| Malde [39] | 7 | 2 | 4 | 1 | 0 | **14** |
| Ellis [29] | 7 | 3 | 3 | 1 | 0 | **14** |
| Cochat [60] | 5 | 3 | 3 | 3 | 0 | **14** |
| Jouvet [32] | 7 | 3 | 3 | 1 | 0 | **14** |
| Lorenz [37] | 7 | 1 | 4 | 1 | 0 | **13** |
| Cochat [59] | 6 | 3 | 3 | 1 | 0 | **13** |
| Watts [58] | 8 | 1 | 3 | 1 | 0 | **13** |
| Jacobs [31] | 6 | 3 | 3 | 1 | 0 | **13** |
| Harambat [18] | 5 | 1 | 5 | 1 | 0 | **12** |
| Hoppe [61] | 5 | 3 | 3 | 1 | 0 | **12** |
| Hoppe [9] | 6 | 3 | 3 | 0 | 0 | **12** |
| Broyer [26] | 5 | 3 | 3 | 1 | 0 | **12** |
| Katz [33] | 7 | 1 | 3 | 1 | 0 | **12** |
| Nolkemper [45] | 6 | 1 | 3 | 1 | 0 | **11** |
| Kemper [34] | 4 | 3 | 3 | 1 | 0 | **11** |
| Narasimhan [44] | 5 | 1 | 3 | 1 | 0 | **10** |
| Watts [54] | 5 | 1 | 3 | 1 | 0 | **10** |
| Binswanger [24] | 4 | 1 | 3 | 1 | 0 | **9** |

**Supplementary Table 4.**

**Study characteristics, patient survival, and graft survival in moderate and low quality studies**

| Reference | Country | Year of publication | Inclusion period | Number of patients | Patient survival | | Graft survival | |
| --- | --- | --- | --- | --- | --- | --- | --- | --- |
| Combined liver kidney transplantation | | | | | | | | |
| Perera [47]^a^ | U.K. | 2009 | 1994-2008 | 9 | 1 y  5 y | 89%  89% | 1 y  5 y | 89%  89% |
| Schmaeschke [52] | Germany | 2017 | 1998-2014 | 13 | 1 y | 100% | 1 y | 77% |
| Gagnadoux [8] | France | 2001 | 1990-2000 | 8 | 5 y | 75% | 5 y | 75% |
| Pratschke [49] | Germany | 1998 | 1988-1997 | 4 | 1 y | 100% | 1 y | 100% |
| Saborio [21] | U.S.A. | 1999 | 1974-1996 | 32 | 6 y  9 y | 55%  40% | 6 y | 56% |
| Lorenzo [38] | Spain | 2006 | 1983-2006 | 2 | 8 y | 100% | 8 y | 100% |
| Watts [58] | U.K. | 1991 |  | 8 | 1 y  3 y | 50%  50% | 1 y  3 y | 50%  50% |
| Narasimhan [44] | India | 2015 |  | 5 | 1 y | 100% |  |  |
| Isolated kidney transplantation | | | | | | | | |
| Saborio [21] | U.S.A. | 1999 | 1974-1996 | 62 | 6 y  9 y | 84%  55% | 6 y  9 y | 44%  33% |
| Sequential liver kidney transplantation | | | | | | | | |
| Brinkert [25] | Germany | 2009 | 1995-2009 | 2 | 1 y | 0% | 1 y | 0% |
| Khorsandi [35] | U.K. | 2016 | 2004-2015 | 5 | 1 y | 80% | 1 y | 80% |
| Sasaki [51] | Japan | 2015 | 2010-2015 | 3 | 1 y | 100% |  |  |
| Kitajima [55] | Japan | 2017 | 1996-2014 | 7 | 1 y | 85.7% | 1 y | 85.7% |
| Narasimhan [44] | India | 2015 |  | 2 | 1 y | 100% | 1 y | 100% |
| Pre-emptive liver transplantation | | | | | | | | |
| Brinkert [25] | Germany | 2009 | 1995-2009 | 4 | 10 y | 100% | 10 y | 100%^b^ |
| Khorsandi [35] | U.K. | 2016 | 2004-2015 | 3 | 1 y | 100% | 1 y | 100% |
| Sasaki [51] | Japan | 2015 | 2010-2015 | 2 | 1 y | 100% | 1 y | 100% |

^a^ Strong quality study.

^b^ One patient received KT after 5.5y.

**Supplementary Table 5.**

**Characteristics of included studies**

| Reference | Country | Year | Inclusion period | Number of patients (Male sex) | Transplantation type | Dialysis (duration)  [median year (range)] | Age at transplantation [median year, (range)] |
| --- | --- | --- | --- | --- | --- | --- | --- |
| Strong quality | | | | | | | |
| Monico [43] | U.S.A. | 2001 | 1968-2000 | 15 (6) | CLKT (7)  KT (8) | 1.4 (0-5.7)  0.3 (0-2.0) | 37 (25-61)  27 (18-52) |
| *Compagnon [16]* | France | 2014 | 1979-2010 | 54 (27) | CLKT (33)  KT (21) | 1.5 (0-72m)  1.3 (0-84m) | 20.3 (1-59)  23.8 (6-58) |
| *Harambat [19]* | France | 2012 | 1979-2009 | 68 | CLKT (53)  SLKT (2)  KT (13) | 62 pts 13.9m (7.2-22.6m^a^) | 7.4 (3.1-13.1^a^) |
| *Bergstrahl [4]* | U.S.A. | 2010 | 1976-2009 | 58 (26) | CLKT (26)  KT (32) | 0.6 (125-426d^a^) | 25.3 (17.4)^b^  30.5 (17.4)^b^ |
| Xiang [22] | China (data from U.S.A.) | 2020 | 1987-2018 | 201 (111) | CLKT (181)  SLKT (20) | 1.4 (0.6-1.8^a^)  0.3 (0.0-1.0^a^) | 20 (8.0-31.5^a^)  11.5 (1.9-27.3^a^) |
| *Cibrik [15]* | U.S.A. | 2002 | 1988-1998 | 190 (105) | CLKT (56)  KT (134) | 31.1 (45)m^b^  37.0 (51)m^b^ | 22.1 (17.8)^b^  35.6 (19.8)^b^ |
| Horoub [30] | Iran | 2019 | 2011-2018 | 24 (17) | CLKT (8)  SLKT (13)  PLT (3) |  | 16 (5-26)  18 (12-32)  4 (3-23) |
| Büscher [27] | Germany | 2015 | 1998-2013 | 11 (7) | CLKT (5)  SLKT (6) | 9 pts HD/PD | 11.8 (8.1-17.8)  1.1 (0.4-1.9) |
| Perera [47] | U.K. | 2009 | 1994-2008 | 9 (5) | CLKT (9) | 8 pts HD/HF 0.4 (19-527d) | 8.6 (1.6-16.7) |
| Moderate quality | | | | | | | |
| Schmaeschke [52] | Germany | 2017 | 1998-2014 | 13 (7) | CLKT (13) |  | 2.8 (1.3-14.2) |
| Nissel [57] | Germany | 2006 | 1989-2006 | 24 (12) | CLKT (23)^c^  SLKT (1) | 1.1 (0.2-13.8) | 11.4 (4.5-15.5) |
| Shapiro [53] | Israel | 2001 | 1989-1998 | 13 (4) | CLKT (7)  SLKT (1)  PLT (3)  KT (2) | 0.5 (0.3-2)  1  1 | 6.5 (1.5-14)  4.5  3.5 (2.5-6)  10.5 (10-11) |
| Quintero-Bernaneu [50] | Spain | 2018 | 2000-2015 | 5 | CLKT (4)  SLKT (1) | HD  HD | 8.2 (3.3-9.4)  1.7 |
| Perera [48] | U.K. | 2011 | 2002-2008 | 4 (4) | PLT (4) |  | (10m-4.5y) |
| Millan [41] | U.S.A. | 2003 | 1994-1998 | 6 (4) | CLKT (6) | HD/PD  (?-16m)  11.8 (2.3)^b^ | (?-20m)  14.8 (3.0m)^b^ |
| Mantel [40] | The Netherlands | 2006 | 1994-2005 | 6 (3) | CLKT (4)  SLKT (2) | HD  HD | 17.5 (1-38) |
| Brinkert [25] | Germany | 2009 | 1995-2009 | 13 | CLKT (7)  SLKT (2)  PLT (4) | HD/PD 1.3 (0.8-2.4)  HD/PD 1.4 (1.1-1.8) | 2.8 (1.4-8.9)  3.7 (2.3-5.0)  4.7 (2.8-9.8) |
| Ozer [46] | Turkey | 2019 | 2017-2018 | 5 (4) | CLKT (1)  SLKT (4) | HD 0.3  HD 3.8 (3m-5.5y) | 10  8 (6-21) |
| Duclaux-Loras [28] | France | 2015 | 1992-2013 | 14 | CLKT (14) | HD/PD 1.6 (0.4-7.6) | 3.7 (1-18.6) |
| Khorsandi [35] | U.K. | 2016 | 2004-2015 | 8 (3) | SLKT (5)  PLT (3) |  | 2 (1-2)  8 (7-14) |
| *Gagnadoux [8]* | France | 2001 | 1990-2000 | 8 (3) | CLKT (8) | 14 (2-42) | 8.2 (1-16) |
| Pratschke [49] | Germany | 1999 | 1988-1997 | 4 | CLKT (4) |  | (21-29) |
| Saborio [21] | U.S.A. | 1999 | 1974-1996 | 94 (?) | KT (62)  CLKT (32) | HD 1.01 (0.9)^b^  HD 1.05 (0.7)^b^ | 14.31 (14) |
| Watts [62] | U.K. | 1991 | 1984-1990 | 24 (13) | CLKT (21)  SLKT (1)  PLT (2) | HD/PD 3.1 (0-9)  6.2 | 21 (4-45)  17  3.5 (2-5) |
| *Kotb [36]* | Egypt | 2019 | 2001-2017 | 5 (1) | CLKT (4)  SLKT (1) | All: HD 5.3 (2.5)^b^ (2.5-9) | 6 (4-10)  15 |
| Sasaki [51] | Japan | 2015 | 2010-2015 | 5 (1) | SLKT (3)  PLT (2) | HD/PD  1 (0.3-5) | 15 (8m-17)  8.5 (7-10) |
| Lorenzo [38] | Spain | 2006 | 1983-2006 | 6 (4) | CLKT (2)  PLT (1)  KT (3) | HD (1-2)  HD | (19-25)  23 |
| Jamieson [20] | U.K. | 2005 | 1984-2004 | 116 (?) | CLKT (100)  SLKT (10)  PLT (6) | All: (0-14.4)  3.2 (3.2)^b^ | (1-38)  16.8^b^ |
| Creput [56] | France | 2003 | 1986-1999 | 8 (4) | CLKT (8) | 7 pts HD | 41.5 (26-57) |
| Milliner [42] | U.S.A. | 1996 | 1948-1997 | 13 | CLKT (4)  KT (8)  PLT (1) |  |  |
| Low quality | | | | | | | |
| Kitajima [55] | Japan | 2017 | 1996-2014 | 7 (4) | SLKT (7) | HD 4 PD 5 | 9 (1-41) |
| Al Riyami [23] | Oman | 2015 | 2000-2013 | 5 (1) | CLKT (2)  SLKT (2)  PLT (1) | HD |  |
| Malde [39] | U.K. | 2011 | 1979-2009 | 3 (1) | KT (1)  SLKT (1)  PLT (1) | HD 5 | 35  31  23 |
| *Ellis [29]* | U.K. | 2001 | 1990-1999 | 6 (2) | CLKT (4)  SLKT (2) | HD 1.08 (0.8-1.8)  HD (2m-2y) | 6.3 (1.7-15.2)  9y2m |
| *Cochat [60]* | France | 1999 | Questionnaire | Tx 33 | CLKT (14)  KT (15)  PLT (4) |  | 6.4 (7.8)^b^  (0.7-21) |
| Jouvet [32] | France | 1998 | 1990-1996 | 7 (2) | CLKT (7) | 0.88 (2m-3.5y) | 7.5 (1-15.7) |
| Lorenz [37] | U.S.A. | 2014 | 1999-2013 | 4 (?) | KT (4) |  | 35.5 (33-67)  42.8 (14.1)^b^ |
| *Cochat [59]* | France | 1995 | 1988-1992 | 26 (?) | CLKT (10)  KT (15)  PLT (1) |  | 29.5 (1-60) |
| Watts [58] | U.K. | 1991 |  | 9 (8) | CLKT (8)  SLKT (1) | HD 6 (5-7)  HD 7 | 26.5 (18-36)  28 |
| *Jacobs [31]* | France | 1975 | Registry | 4 (?) | KT (4) | 24m^b^ |  |
| *Harambat [18]* | France | 2010 | 1993-2008 | 72 | CLKT (41)  PLT (2)  KT (29) |  |  |
| *Hoppe [61]* | Germany | 2005 | 1994-2000-2004 | 22 (?) | CLKT (9)  SLKT (2)  PLT (5)  KT (6) |  |  |
| *Hoppe [9]* | U.S.A. | 2003 | Survey 1997 | Tx 48 | CLKT (12)  KT (27) |  |  |
| *Broyer [26]* | France | 1996 | 1990-1995 | 4 (1) | CLKT (4) | HD 1.2 (0.2-4) | 3.1 (0.9-13) |
| Katz [33] | Israel | 1992 |  | 8 (?) | KT (8) | 0,8 (5m-1.5y) | 1.0 (7m-8y) |
| Nolkemper [45] | Germany | 2000 |  | 4 (?) | PLT (4) |  | 4.7 (2.8-9.8) |
| Kemper [34] | Germany | 1998 |  | 4 (?) | PLT (4) |  | 4.7 (2.8-9.8) |
| Narasimhan [44] | India | 2015 |  | 7 (2) | CLKT (5)  SLKT (2) |  | 15 (9-47) |
| Watts [54] | U.K. | 1988 |  | 6 (3) | KT (6) |  |  |
| *Binswanger [24]* | Switzerland | 1986 |  | 7 (6) | KT (7) | HD/PD 2 (0.5-7) | 26 (21-53) |

Cursive studies: PH diagnosis based on clinical characteristics/metabolites in some of the included patients.

Pts: patients; ?: not reported.

^a^ Interquartile range.

^b^ mean (SD).

^c^ one patient had SLKT (3 months in between), not reported which patient.

**Supplementary Table 6.**

**Transplantation outcomes of patients with primary hyperoxaluria type 1**

| Reference | Type of Tx | Median follow-up (range) | Outcome at last follow-up | | | | | |
| --- | --- | --- | --- | --- | --- | --- | --- | --- |
|  |  |  | **Functioning graft** | | **Graft loss due to^a^** | | | **Death** |
|  |  |  | **Number of patients** | **eGFR [ml/min/1.73m^2^] mean (SD)** | **Recurrence of oxalosis** | **Rejection** | **Other reason** |  |
| Strong quality | | | | | | | | |
| Monico [43] | CLKT  KT | 2.1y (0.24-13.9y)  1.8y (0-20.2y) | 4 (57.1%)  3 (37.5%) | 1.2 (0.3)  1.7 (0.6) | 2 | 3  2 | 1 | 2  1 |
| Compagnon [16] | CLKT  KT | 80m^b^ (0.1-239m)  46m^b^ (2.3-281m) | 27 (82%)  2 (10%) |  | 11 | 3  4 | 1  2LT  4 | 22% (15y)  40% (15y) |
| Harambat [19] | CLKT  SLKT  KT | 5y | LKT: 76%  14% |  |  |  |  |  |
| Bergstralh [4] | CLKT  KT | 1.3y (0.3-3.2^c^)  9.7y (6.3-14^c^) | 5 y (48%)  5 y (45%)  All last FU: 32 |  | All last FU: 10 | All last FU: 5 | All last FU: 11 | 33% (5y)  0% (5y)  All last FU: 12 |
| Xiang [22] | CLKT  SLKT |  | 5 y (78.1%)  5 y (85.0%) |  | 1  0 | 0  1 | 14, 7LT  2 | 23% (10y)  16% (10y) |
| Cibrik [15] | CLKT  KT |  | Adjusted DC (8y)  CLKT 76%  KT 47.9% (p<0.01) |  |  |  |  | Adjusted (8y)  CLKT and KT ≈63% |
| Horoub [30] | CLKT  SLKT  PLT |  | 5 (62.5%)  9 (69.2%)  2 (66.7%) |  |  | 0  0  1 | 3  4 | 3  4  1 |
| Büscher [27] | CLKT  SLKT | 11.6y (0.3-16.3)  2.4y (1.3-12.7) | 4 (80%)  5 (83%) |  |  |  | 1LT  1LT | 1  1 |
| Perera [47] | CLKT | 7.3y (14-112m) | 8 (89%) | 58.18 |  |  | 1 | 1 |
| Moderate quality | | | | | | | | |
| Schmaeschke [52] | CLKT | 4.8y (1.0-10.8) | 10 (76.9%) | 63.4 (21.6) |  |  | 3 | 0 |
| Nissel [57] | CLKT  SLKT | All: 5.7y (3.2)^b^ (1-12) | 23 (100%)  1 (100%) | All: 67 (23) |  |  |  | 0  0 |
| Shapiro [53] | CLKT  SLKT  PLT  KT | 1.3 y (0-4)  4.5 y  3 y (3-3.5)  6.2 y (2-10.3)  Dx to FU 2.5y (0.6m-19y) | 5(62.5%)  1(100%)  3(100%)  0 (0%) | 83 (6.6)  55  87.3 (4.0) | 0 | 0 | 1LT | 2  0  0  1 |
| Quintero-Bernaneu [50] | CLKT  SLKT |  | 2 (50%)  1 (100%) |  |  | 1 | 1 | 0  0 |
| Perera [48] | PLT | 5.3y (24-84m) | 3 (75%) | 72 |  |  | 1 | 0 |
| Millan [41] | CLKT | 6.4y (1.7)^b^ (3.9-8.1) | 6 (100%) | 99.2 (27.4) | 0 | 0 | 0 | 0 |
| Mantel [40] | CLKT  SLKT | All: 23m (0.3-117m) | All: 3 (50%) |  | 1 |  | 2LT | 1 |
| Brinkert [25] | CLKT  SLKT  PLT | 3.0y (0.2-10.2)  6m (1-11m)  11.5y (11.3-12.1) | 7 (100%)  0 (0%)  4 (100%) | 89.3 (24)  104.7 (14.4) |  |  | 2 | 0  2  0 |
| Ozer [46] | CLKT  SLKT | 1.0y  1.1y (2m-19m) | 1 (100%)  2 (50%) | 125^d^  51.1 (0.5)^d^ | 1 |  | 1^e^ | 0  1^e^ |
| Duclaux-Loras [28] | CLKT | 4.1y (0.5-13.3) | 11 (78.6%) | 79.4 (36.0) |  |  | 3 | 0 |
| Khorsandi [35] | SLKT  PLT | 4.0y (1.5-8.3)  6.9y (2.5-7.3) | 4 (80%)  3 (100%) (1 PLT required additional KT) | 2 pts: 55 |  |  | 1 | 1  0 |
| Gagnadoux [8] | CLKT | 6y (5-11) | 6 (75%) | 111.2 (18.6) |  |  | 2 | 2 |
| Pratschke [49] | CLKT | 28.6m^b^ (8-80)^f^  PH: 18-42m | 4 | 2.4 (1.4) mg/dl^g^ | 0 | 0 | 0 | 0 |
| Saborio [21] | CLKT  KT |  | 6 y 65%  6 y 50% |  |  |  |  | 16% (6y)  35% (6y) |
| Watts [62] | CLKT  SLKT  PLT | 0.7y(0.5-41m)  1.3y  1.6y(18-21m) | 14 (67%)  0  2 (100%) | 61.6 (23.6)  Stable |  |  | 3 | 4  1  0 |
| Kotb [36] | CLKT  SLKT | All: 5y survival calculated | 2 (50%)  0 |  |  |  |  | 2  1 |
| Sasaki [51] | SLKT  PLT | 0.42m (2-32)  1.33m (12-20) | 2 (66.7%)  2 (100%) | 60.9 (6.9) |  |  | 1LT | 1  0 |
| Lorenzo [38] | CLKT  PLT  KT | 8y, 12y  11y  1.2y (0.3-7) | 2 (100%)  1 (100%)  0 | 51.7 (28.2)  65 | 0  0  3 |  |  | 0  0  3 |
| Jamieson [20] | CLKT  SLKT  PLT |  | All: 66 (56.9%) | 35-70^d^ | 7 |  | All: 6, 10LT | All: 27 |
| Creput [56] | CLKT | 5y (14m-14y)^f^ | 5 (62.5%) | 137 µmol/l^g^ | 0 | 0 | 3 | 3 (37.5%) |
| Milliner [42] | CLKT  KT  PLT | All^f^: 10.4y (8.7)^b^ (<1-29) | 8 renal^f^  4 liver^f^ |  | All: 3^f^ | All: 1^f^ | All: 2^f^ | All: 6^f^ |
| Low quality | | | | | | | | |
| Kitajima [55] | SLKT | 7.9y (0.17-19.7) | 5 (71.4%) | 48.4 (16.7) |  | 1 |  | 1 |
| Al Riyami [23] | CLKT  SLKT  PLT | 11y (9-13)  8.3y (7.5-9)  4y | 1 (50%)  1 (50%)  1 (100%) |  | 1 |  |  | 0  1  0 |
| Malde [39] | KT  SLKT  PLT | 5y  4y  7y | 0  1 (100%)  1 (100%) |  | 1  0 | 0 | 0 | 0  0  0 |
| Ellis [29] | CLKT  SLKT | 6.5 m (0.7-10m)  7y5m | 3 (75%)  1 (50%) | 95, 55, 20  16.9 | 1 |  |  | 1  1 |
| Cochat [60] | CLKT  KT  PLT |  | 11(78.6%)  4 (26.7%)  3 (75%, 1 pts required additional KT) |  |  |  | 1  3 | 2  8  1  All: 33% died |
| Jouvet [32] | CLKT | 2.4y (0.4-6.3) | 6 (85.7%) | 84 (17.3)^h^ |  |  | 1LT | 1 |
| Lorenz [37] | KT | 5.2y (0.2-13.9) | 3 (75%) |  | 1 |  |  | 0 |
| Cochat [59] | CLKT  KT  PLT | >1y | 7 (70%)  1 (6.7%)  1 (100%) |  |  | 1 | 9 | 2  5  0 |
| Watts [58] | CLKT  SLKT | 1.9y (0-4.2)  1.2y | 4 (50%)  0 | 2 pts 74.5 (26.5) | 1 | 1 |  | 4  1 |
| Jacobs [31] | KT | 18, 45m | 2 (50%) |  | 2 |  |  | 1 |
| Harambat [18] | CLKT  PLT  KT | 5y (2d-15.7y) | 3 (10.3%) |  | 24 |  | 2 |  |
| Hoppe [61] | CLKT  SLKT  PLT  KT | 6m-11y  7-16y | 5 (55.6%)  1 (50%)  4 (80%)  1 (16.7%) |  | 2  3 |  | 2LT  1  2 | 1  0  1  0 |
| Hoppe [9] | CLKT  KT |  | 13 (48.1%) |  | 8 |  | 6 | 2 |
| Broyer [26] | CLKT | 2y (3m-5y) | 4 (100%) |  | 0 | 0 | 0 | 0 |
| Katz [33] | KT | 7.5y (6-10) | 2 (25%) | 102 (17.2) |  |  | 2 | 4 |
| Nolkemper [45] | PLT | 3.2y (3-4.7) | 4 (100%) |  |  |  |  | 0 |
| Kemper [34] | PLT | 0.5y (4-24m) | 4 (100%) |  | 0 | 0 | 0 | 0 |
| Narasimhan [44] | CLKT  SLKT | 2y1m (15-36m)  16, 15m | 4 (80%)  2 (100%) |  |  |  | 1 | 0  0 |
| Watts [54] | KT | 0.8m (5-21m) | 2 (33%) | 49 (8) | 3 |  | 1 | 0 |
| Binswanger [24] | KT |  | 0 |  |  | 3 | 4 | 3 |

Pts: patients; LT: liver transplant; ?: not reported.

^a^ kidney graft loss if not further specified.

^b^ mean (SD).

^c^ interquartile range.

^d^ ml/min.

^e^ died before KT.

^f^ including non-PH1 patients and/or non-transplanted patients.

^g^ creatinine.

^h^ creatinine clearance µM.
